# A Practical Approach to Predicting Surgical Site Infection Risk Among Patients Before Leaving the Operating Room

**DOI:** 10.7759/cureus.42085

**Published:** 2023-07-18

**Authors:** Michael S Woods, Valerie Ekstrom, Jonathan D Darer, Jacqueline Tonkel, Isabell Twick, Bruce Ramshaw, Aviram Nissan, Dan Assaf

**Affiliations:** 1 Global Chief Medical Officer, Caresyntax Corp, Boston, USA; 2 Director, Clinical Affairs, Caresyntax Corp, Boston, USA; 3 Medical and Innovation Director, Health Analytics LLC, Maryland, USA; 4 Senior Vice President, Client Engagement Clinical Transformation, Caresyntax Corp, Boston, USA; 5 Data Scientist, Caresyntx Corp, Boston, USA; 6 Co-Founder and CEO, CQInsights PBC, Knoxville, USA; 7 Department of General and Oncological Surgery – Surgery C, Chaim Sheba Medical Center, Tel Aviv, ISR

**Keywords:** surgical site infection (ssi), operative safety, predictive model, pre-operative risk factors, intraoperative risk factors

## Abstract

A surgical site infection (SSI) prediction model that identifies at-risk patients before leaving the operating room can support efforts to improve patient safety. In this study, eight pre-operative and five perioperative patient- and procedure-specific characteristics were tested with two scoring algorithms: 1) count of positive factors (manual), and 2) logistic regression model (automated). Models were developed and validated using data from 3,440 general and oncologic surgical patients. In the automated algorithm, two pre-operative (procedure urgency, odds ratio [OR]: 1.7; and antibiotic administration >2 hours before incision, OR: 1.6) and three intraoperative risk factors (open surgery [OR: 3.7], high-risk procedure [OR: 3.5], and operative time OR: [2.6]) were associated with SSI risk. The manual score achieved an area under the curve (AUC) of 0.831 and the automated algorithm achieved AUC of 0.868. Open surgery had the greatest impact on prediction, followed by procedure risk, operative time, and procedure urgency. At 80% sensitivity, the manual and automated scores achieved a positive predictive value of 16.3% and 22.0%, respectively. Both the manual and automated SSI risk prediction algorithms accurately identified at-risk populations. Use of either model before the patient leaves the operating room can provide the clinical team with evidence-based guidance to consider proactive intervention to prevent SSIs.

## Introduction

Surgical site infections (SSIs) are the most common post-surgical complication, and in the United States, 2-4% of inpatient surgical procedures are associated with SSIs and are the leading cause of unplanned hospital readmissions among surgical patients [1]. SSIs represent an estimated $3.3 billion in US healthcare costs [2]. While substantial reductions in SSIs have been achieved through infection controls, sterilization methods, maintaining normothermia, perioperative glycemic control, and antimicrobial prophylaxis [2,3], SSIs continue to be a major source of morbidity and cost.

Identifying patients at high-risk for SSIs is an important component of SSI reduction programs. Identifying high-risk SSI patients can support surgical consent, the application of intensive prevention measures, and increased post-operative monitoring. Accurate SSI risk prediction can also enable risk-adjusted SSI comparative performance reporting for the purpose of transparency and improvement [4].

While there are many published procedure-specific SSI risk calculators, those intended to be used across surgical procedures include the Surgical Site Infection Risk Score (SSIRS) [4], JSS-SSI Risk Scoring Tool [5], and the American College of Surgeons - National Surgical Quality Improvement Program (ACS-NSQIP) risk score [6]. The SSIRS is composed of 13 items and has a reported area under the curve (AUC) statistic of 0.8, the JSS-SSI tool is composed of five items and has a reported AUC of 0.66, and the ACS-NSQIP is composed of 21 items with a reported AUC for SSI ranging from 0.73-0.82 [7,8]. The most widely used of these tools, the ACS-NSQIP, with 21 items to enter is relatively onerous to use, especially in the standard workflow of the operating room (OR), one of the most complex, high-stress environments in healthcare. All three of the aforementioned models require the use of an actual calculator embedded with specific variable weights that may not be easy to access in all surgical environments. With the goal of incorporating SSI risk prediction into routine surgical operations, we sought to develop and evaluate a straightforward predictive instrument based on commonly available pre-operative and intraoperative data to identify high-risk SSI patients before leaving the OR.

## Materials and methods

In this study, we developed an SSI predictive model (SSI-PM) based on review of SSI literature and performed a retrospective, observational, split sample cohort study to assess the SSI-PM’s predictive accuracy in a real-world population of surgical patients using data extracted from an electronic health record (EHR).

Risk factor identification

To identify candidate SSI risk factors, a PubMed narrative literature review was performed using search terms related to SSI events and risk. The review identified 14 risk factors that were a) independently associated with statistically significant increased SSI risk and b) based on data that is readily available in EHR data or easily reported from data collected from the surgical theater. Eight pre-operative risk factors were identified: low albumin (present if <3.5) [8], American Society of Anesthesiologists Physical Status Classification (ASA) score (present if ≥ 3) [8,9], abnormal body mass index (BMI) (present if <20 or >35 kg/m^2^) [9], comorbid diabetes mellitus [8,9], procedure urgency (present if trauma or emergency) [10], prophylactic antibiotic administered <120 minutes before incision [9], current tobacco use [8,9], and current steroid use [8]. Six intraoperative risk factors were identified: open surgery [11], blood loss (present if ≥600 mL) [10], perioperative blood transfusion [10], prolonged operative time (present if >2 hours) [9], high-risk procedure (present if colorectal or small bowel procedure) [8,10], and wound classification [8] (Table 1). Though wound classification is readily reportable in the OR, it was not available in the EHR and was excluded from the analysis, leaving 13 in the final model.

**Table 1:** Risk factors identified as predictive of a surgical site infection *Wound class was not available in the analytical dataset and excluded as a risk factor in this analysis. ASA, American Society of Anesthesiologists physical classification score; BMI, body mass index

Risk category	Risk factor	Source
Perioperative		
Albumin level	<3.5 (vs. ≥3.5)	[8]
ASA score	≥3 (vs. <3)	[8,9]
BMI	<20 or >35 kg/m^2 ^(vs. 21-35)	[9]
Comorbid diabetes mellitus	Yes (vs. no)	[8,9]
Procedure urgency	Emergency or trauma (vs. any other urgency level)	[10]
Pre-procedure administration of antibiotics	>120 min before incision (vs. ≤120 min)	[9
Tobacco use	Current (vs. never/former)	[8,9]
Steroid use	Current (vs. not current)	[8]
Intraoperative		
Blood loss	≥600 mL (vs. <600 mL)	[10]
Blood transfusion required	Yes (vs. no)	[10]
Operative time	>2 hours (vs. ≤2 hours)	[9]
High-risk procedure	Colorectal or small bowel procedure (vs. any other procedure)	[8,10]
Wound class*		[8,9]

Patient selection and inclusion criteria

The analytical dataset was derived from all patients undergoing a select set of general and oncological surgical operations including colorectal, small bowel, gastroesophageal, breast, diagnostic, hernia, and biliary tract procedures between September 2017 and September 2019 in the Department of General and Oncological Surgery at The Chaim Sheba Medical Center, Ramat Gan, Israel. Only the first procedure conducted during the study period for an individual patient was eligible for inclusion in the analysis. The final dataset included 3,440 unique surgical patients and procedures. SSIs were prospectively recorded at the time of diagnosis and verified using an EHR-based, mandatory review process prior to discharge.

Statistical analysis and modeling

Descriptive statistics were calculated on the patient cohort using prevalence and percentage values for categorical variables and means and standard deviations for continuous variables. The prevalence of each risk factor and its association with SSIs was assessed based on the training dataset containing 2,048 surgical patients. Variable distribution and skew are presented by median and interquartile range. Group comparisons were performed using Student’s T-test for continuous variables. Comparisons for categorical variables were calculated using the χ2 test or Fisher's exact test as appropriate. Unadjusted odds ratios (ORs) for individual risk factors’ association with SSI events were also calculated.

Predictive accuracy was assessed using two models: a) simple count of risk factors (score: 0-13) and b) multivariate logistic regression model predicting SSI outcomes in a population of surgical events. To develop and validate each predictive method, the dataset was randomly divided (70%/30%) into training (n= 2,408 procedures) and test (n=1,032 procedures) datasets. Training of the logistic regression model was performed on the dataset using generalized linear models with a logit link function. Risk factors served as inputs for the model, and SSI occurrence served as the output. Measures of model performance for both predictive models were derived from application of the models in the test dataset. Overall, model performance was based on AUC of the receiver operating characteristic (ROC) curve, which plots model sensitivity versus model specificity.

To calculate different levels of model sensitivity, specificity, and positive and negative predictive value, a series of progressively higher score threshold (or cut-off) levels were selected. In this approach, individuals above that score threshold are assumed to be at a high risk of SSI and then compared against the actual presence of SSI as measured in the EHR data. The high-risk cut-off scores for the simple count of positive items were the count of positive risk factors, ranging from the lowest threshold requiring one or more risk factors to be considered high risk up to the highest threshold measured requiring at least eight positive risk factors to be considered at high-risk SSI. Cut-off risk probabilities for the logistic regression model were matched to the ones of the simple model based on equal sensitivity values.

To summarize the association between the observed and predicted binary classifications in a single metric, F1 score and Matthews’s correlation coefficient (MCC) were calculated for each high-risk cut-off. Though the F1 score, the harmonic mean of the positive predicted value and sensitivity, is the more commonly used measure, it ignores correctly classified negative examples. MMC, on the other hand, is regarded as a superior measure as it describes all four confusion matrix categories (true positives, false positives, true negatives, false negatives). The MCC returns values between -1 and +1, whereas a coefficient of +1 represents a perfect prediction, 0 is no better than random chance, and -1 indicates complete disagreement. Statistical testing and regression modeling using the glm library was performed in R software [12].

Ethics approval

The Tel Hashomer Helsinki Committee approved the research and waived the requirement to obtain informed consent (SMC-6411-19). All methods were performed in accordance with privacy and confidentiality guidelines and regulations.

## Results

Among the 3,440 surgical patients, 54.7% were female, with a mean age of 52.0 years (SD: 18.3 years). Common patient conditions included neoplasm (18.3%), diseases of the circulatory system (8.6%), diseases of metabolism (8.4%), and diseases of the digestive system (8.0%). All other patient conditions were reported at a frequency of less than 5%. The median number of medications used was 1 (interquartile range [IQR]: 0-4) (Table 2).

**Table 2:** Overview of the study population ICU, intensive care unit; n.s., not statistically significant; SD, standard deviation; SSI, surgical site infection

	Total (N=3,440)		Training (N=2,408)		Test (N=1,032)		
	N	%	N	%	N	%	p
Demographics							
Female	1,883	54.7	1,343	55.8	540	52.3	n.s.
Age, years (mean/SD)	52.0	(18.3)	51.9	(18.3)	52.3	(18.3)	n.s.
Comorbidities							
Neoplasms	631	18.3	438	18.2	193	18.7	n.s.
Circulatory diseases	297	8.6	213	8.8	84	8.6	n.s.
Metabolic diseases	289	8.4	195	8.1	94	9.1	n.s.
Digestive diseases	275	8.0	186	7.7	89	8.6	n.s.
Mental diseases	85	2.5	57	2.4	28	2.7	n.s.
Tissue diseases	74	2.2	57	2.4	17	1.6	n.s.
Genitourinary diseases	59	1.7	43	1.8	16	1.6	n.s.
Nervous system diseases	59	1.7	39	1.6	20	1.9	n.s.
Eye diseases	51	1.5	36	1.5	15	1.5	n.s.
Respiratory diseases	42	1.2	32	1.3	10	1.0	n.s.
Medications							
Number of medications, median (IQR)	1 (0–4)		1 (0–4)		1 (0–4)		n.s.
Surgery type							
Hernia, n (%)	551	16.0	369	15.3	182	17.6	n.s.
Gastroesophageal	515	15.0	368	15.3	147	14.2	n.s.
Colorectal	440	12.8	319	13.2	121	11.7	n.s.
Biliary tract	418	12.2	304	12.6	114	11.0	n.s.
Breast	377	11.0	256	10.6	121	11.7	n.s.
Appendectomy	320	9.3	237	9.8	83	8.0	n.s.
Diagnostic	226	6.6	160	6.6	66	6.4	n.s.
Small bowel	190	5.5	125	5.2	65	6.3	n.s.
Abdomen/retroperitoneum	83	2.4	54	2.2	29	2.8	n.s.
Anorectal	80	2.3	51	2.1	29	2.8	n.s.
Gynecological	50	1.5	32	1.3	18	1.7	n.s.
Hepatic	31	0.9	19	0.8	12	1.2	n.s.
Outcomes							
ICU admission, n (%)	63	1.8	39	1.6	24	2.3	n.s.
Length of stay (mean, SD)	5.1	(10.9)	5.0	(9.8)	5.3	(13.2)	n.s.
30-day readmission, n (%)	43	1.2	29	1.2	14	1.4	n.s.
30-day mortality, n (%)	33	1.0	24	1.0	9	0.9	n.s.
SSI, n (%)	163	4.7	107	4.4	56	5.4	n.s.
Superficial incisional SSI, n (%)	145	4.2	93	3.8	50	4.8	n.s.
Deep incisional SSI, n (%)	14	0.4	10	0.4	4	0.4	n.s.
Organ-space SSI	6	0.2	4	0.2	2	0.2	n.s.

Most of the surgical procedures were performed laparoscopically (51.9%), and 23.3% were urgent. The most common surgical procedure was hernia repair (16.0%), followed by gastroesophageal (15.0%), colorectal (12.8%), biliary tract (12.2%), and breast (11.0%) procedures. The average length of stay was 5.1 days (SD: 10.9), with only 1.8% of procedures resulting in an intensive care unit (ICU) admission. The 30-day mortality and readmission rates were 1.2% and 1.0%, respectively. The SSI event rate was 4.7%, affecting 163 individuals (Table 2).

The 70% sample randomly assigned to the training dataset were comparable to the 30% assigned to the validation sample, with no statistically significant differences in demographic characteristics, comorbidities, surgery type, or surgical outcomes, including SSI rate, length of stay, ICU admission rate, 30-day readmission rate, or 30-day mortality rate (Table 2).

SSI risk factors

Prior to adjustment, the most common factors significantly and positively associated with SSI event risk were open (vs. laparoscopic) surgery (46.8%), operative time > 2 hours (43.7%), ASA score ≥ 3 (42.5%), antibiotic administration greater than 2 hours of incision (25.7%), abnormal BMI (25.4%), procedure urgency (22.9%), current tobacco use (21.6%), and high-risk procedure (18.4%). The least commonly reported risk factors were requiring a blood transfusion (6.9%), current steroid use (2.8%), intraoperative blood loss ≥ 600 mL (2.3%), and comorbid diabetes mellitus (1.9%) (Table 3).

**Table 3:** Risk factors and SSI event rates among the 2,408 operations included in the training dataset *High-risk procedure includes colorectal or small bowel surgery ASA, American Society of Anesthesiologists physical classification score; BMI, body mass index; SSI, surgical site infection

Risk factor	n	%	Unadjusted odds ratio	95% CI	Adjusted odds ratio	95% CI
Pre-operative						
Albumin level	184	7.6	3.4	1.9–5.7	1.5	0.8–2.6
ASA score	1023	42.5	2.3	1.5–3.6	1.5	0.9–2.4
BMI	612	25.4	0.6	0.3–1.0	1.2	0.6–2.1
Diabetes mellitus (type 1 or 2)	46	1.9	1.7	0.3–5.4	1.2	0.3–4.4
Procedure urgency	552	22.9	1.9	1.2–3.0	1.7	1.0–2.8
Pre-procedure antibiotic administration	618	25.7	1.3	0.8–2.0	1.6	1.0–2.7
Tobacco use	520	21.6	0.6	0.3–1.1	0.8	0.5–1.5
Steroid use	67	2.8	1.5	0.4–4.2	0.7	0.2–2.2
Intraoperative						
Open surgery	1,127	46.8	7.1	4.1–13.0	3.7	2.1–6.7
Blood loss	55	2.3	4.3	1.7–9.5	0.9	0.4–2.4
Blood transfusion	167	6.9	5.9	3.5–9.6	1.2	0.7–2.3
Operative time	1052	43.7	6.1	3.6–10.9	2.6	1.4–4.8
High-risk procedure*	444	18.4	9.6	6.1–15.2	3.5	2.1–5.9

After adjustment for all other risk factors, open surgery had the strongest association with SSI events (OR: 3.7; 95% CI: 2.1-6.7), followed by high-risk procedure (OR: 3.5; 95% CI 2.1-5.9), operative time > 2 hours (OR: 2.6; 95% CI: 1.4-4.8), procedure urgency (OR: 1.7; 95% CI: 1.0-2.8), and pre-procedure antibiotic administration greater than 2 hours before incision (OR: 1.6; 95% CI: 1.0-2.7). The assignment of colorectal and small bowel procedures to the high-risk procedure category was confirmed by a comparison of each procedure type with the likelihood of an SSI event. Colorectal (OR: 6.6; 95% CI: 4.2-10.2) and small bowel (OR: 4.6; 95% CI: 2.5-8.1) surgery were positively associated with SSI risk, with each having an SSI event rate of 14.4% (Table 4).

**Table 4:** Distribution of procedure types, SSI event rate, and unadjusted odds ratios and 95% CI included in the 2,048 operations in the training dataset SSI, surgical site infection

Procedure	n	%	SSI event (%)	Unadjusted OR	95% CI	p
Colorectal	319	13.2	14.4	6.6	4.2–10.2	<0.001
Small bowel	125	5.2	14.4	4.6	2.5–8.1	<0.001
Abdomen retroperitoneum	54	2.2	7.4	1.9	0.5–5.4	NS
Gynecology	32	1.3	6.2	1.6	0.2–6.4	NS
Diagnostic	160	6.6	3.1	0.7	0.2–1.8	NS
Other	114	4.7	2.6	0.6	0.1–1.9	NS
Hernia	369	15.3	1.9	0.4	0.2–0.9	<0.05
Appendectomy	237	9.8	1.7	0.4	0.1–1.0	NS
Biliary tract	304	12.5	1.3	0.3	0.1–0.8	<0.01
Gastroesophageal	368	15.3	0.8	0.2	0.0–5.1	<0.001
Breast	256	10.6	0.8	0.2	0.0–0.6	<0.01
Anorectal	51	2.1	0	0	0–1.7	NS
Hepatic	19	0.8	0	0	0–5.1	NS

No other surgery type included had a positive association with SSI risk, defined by unadjusted OR. The remaining risk factors including low albumin level, ASA score, abnormal BMI, comorbid diabetes mellitus, current tobacco use, current steroid use, intraoperative blood loss, and intraoperative blood transfusion required were not associated with SSI event risk after OR adjustment (Table 3).

Predictive accuracy

**Figure 1:**
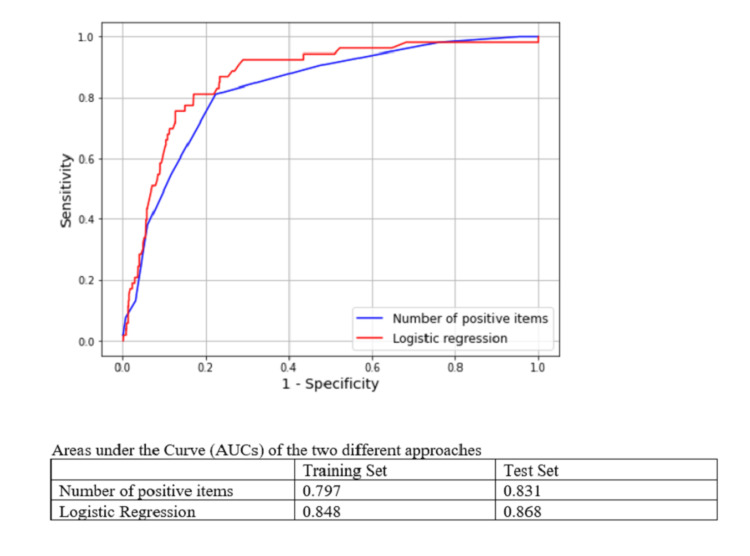
Receiver operator characteristic (ROC) curves for the two versions of the SSI prediction models

Model performance statistics including ROC curves were calculated on the test dataset containing 1,032 surgical patients (Table 5, Figure 1).

**Table 5:** Performance of the SSI prediction models based on the number of positive items and logistic regression applied to the 1,032 operations included in the validation sample MCC, Matthews correlation coefficient; NPV, negative predictive value; PPV, positive predictive value; SSI, surgical site infection

Cut-score threshold	Sensitivity	Specificity	PPV	NPV	Patients classified as high risk		F1 score	MCC
					N	%		
SSI predictive model: count of positive items								
≥1	100	4.5	5.6	100	988	95.7	0.102	0.049
≥2	98.1	23.8	6.5	99.6	798	77.3	0.122	0.116
≥3	90.6	52.4	9.3	99.0	514	49.8	0.169	0.190
≥4	81.1	77.5	16.3	98.7	263	25.5	0.272	0.297
≥5	54.7	88.3	20.1	97.3	144	14.0	0.294	0.274
≥6	37.7	94.0	25.3	96.5	79	7.7	0.303	0.263
≥7	13.2	96.8	18.4	95.4	38	3.7	0.154	0.118
≥8	7.5	99.3	36.4	95.2	11	1.1	0.125	0.147
SSI predictive model: multivariate logistic regression								
≥0.000262	100.0	0.0	5.1	100	1032	100.0	0.098	0.000
≥0.000953	98.1	31.8	7.2	99.7	720	69.8	0.135	0.144
≥0.003140	90.6	72.1	15.0	99.3	321	31.1	0.257	0.299
≥0.005409	81.1	82.9	20.5	98.8	210	20.3	0.327	0.351
≥0.012850	54.7	91.5	25.9	97.4	112	10.9	0.352	0.328
≥0.016896	39.6	94.3	27.3	96.6	77	7.5	0.323	0.285
≥0.027346	13.2	98.6	33.3	95.5	21	2.0	0.189	0.184
≥0.032325	5.7	99.1	25.0	95.1	12	1.2	0.092	0.098

 

The AUCs for the predictive models based on count of risk factors and logistic regression were 0.83 and 0.87, respectively. For both models, the MCC - a measure of association between the predicted group (high versus low risk) and the actual group (SSI versus no SSI) - was largest at a sensitivity of 80% (= the models identify 80% of all patients with an SSI event). However, the MCC score was higher for the logistic regression model with an MCC of 0.35 than for the count of positive items model, with MCC = 0.30, at 80% sensitivity. In line with this, other accuracy metrics also varied between the two models. At a sensitivity of 80%, the manual score requires the presence of four or more risk factors; it has a specificity of 77.5%, a positive predictive value of 16.3%, and a negative predictive value of 98.7%; it categorizes 25.5% of patients as high-risk for SSI who might benefit from aggressive management including prophylaxis; and 74.5% of patients as low risk who can be managed with greater efficiency and fewer interventions. Similarly, setting the automated (regression-based) model output at a sensitivity of 80% and the model specificity is 82.9%, has a positive predictive value of 20.5% and a negative predictive value of 98.8%, and categorizes 20.3% of patients as high-risk for SSI and 79.7% as low-risk.

## Discussion

Despite international efforts to reduce infections, SSIs continue to be a substantial source of clinical morbidity and economic burden. Focusing surveillance and prevention efforts on high-risk patients has the potential to support cost-effective SSI reduction. Existing general SSI prediction models have limited predictive accuracy, can require substantial data entry, and require data entry into a pre-programmed calculator commonly external to the day-to-day workflow of clinicians. Anticipating the potential for surgical teams to have limited access to real-time analytics, we developed and validated two different predictive algorithms derived from 13 evidence-based risk factors readily available to the surgical team, a manual count, and a regression-based algorithm requiring real-time computation. The manual scoring algorithm has the advantage of being easy to integrate into non-electronic clinical workflows, whereas the automated algorithm requires real-time analytical capabilities but provides a more accurate risk assessment. Both SSI risk models had comparable or slightly better AUC curves compared to other reported SSI tools including the SSIRS, JSS-SSI, and ACS-NSQIP.

In our analysis, after adjusting for all present risk factors, eight of the proposed 14 risk factors including BMI, diabetes mellitus, tobacco use, albumin level, ASA score, intraoperative blood loss, intraoperative blood transfusion, and concomitant steroid use were not associated with SSI risk. In systematic reviews of SSI risk factors, inconsistent associations between potential risk factors and SSI event risk have been noted [9,13]. Reasons for inconsistent results regarding risk factor predictive capability can include variable definition, specific surgeries included in a study, and variations in study characteristics. Although these eight factors were not associated with increased risk for SSI in this study, the published literature provides compelling evidence that they can be associated with increased risk and thus should be included in further research and development of the SSI-PM.

The incorporation of SSI-PM into routine surgical care has the potential to support and augment existing efforts to reduce SSIs. The impact of traditional surgical safety checklists has been inconsistent [14]. While overall SSIs for inpatient procedures in the United States decreased by 16% from 2010-2015, initial reports of one of the more prominent U.S. efforts to reduce SSIs using checklists, the Surgical Care Improvement Program (SCIP) (2010-11), was ineffective at reducing SSIs [15,16]. Barriers to successful surgical checklist implementations and achieving reductions in SSIs include confusion regarding how to properly use a checklist, challenges in workflow efficiency, general surgeon resistance to change, concerns about legal implications of signing a checklist form, failure to perform safe surgical practices despite documentation of completion, variations in surgical team competency, and the importance of a culture of safety [15-18]. Using SSI prediction tools such as the SSI-PM has the potential to increase motivation and focus surgical staff to adhere to checklist procedures for those at greatest risk.

Considering the need for fiscal responsibility in healthcare, SSI risk prediction has the potential to optimize the impact of limited resources, enabling more targeted use of SSI prevention measures such as antibiotics and negative pressure wound therapy. Additionally, almost all SSIs resulting in readmission occur after patient discharge [19], and delays in detection and treatment can result in serious consequences. Moreover, patients at high-risk for SSI are excellent candidates to receive more intensive prevention such as the use of negative pressure wound therapy [20] as well as enhanced post-operative monitoring to enable early SSI detection and treatment [21,22].

The success of SSI risk identification and reduction tools will continue to be limited until they seamlessly integrate into clinical workflow and provide simple, actionable analytics tied to clear clinical decision-making guidance at the point of care. Future research will seek to assess the SSI model in different surgical populations and evaluate impact in real-world surgical settings. To reduce barriers to adoption, advanced versions of the SSI-PM regression model should minimize data entry, auto-populate with risk factors recorded in the EHR, and enable real-time guidance to reduce SSI.

Limitations

In keeping with a real-world evidence generation approach, the identification and selection of potential risk factors was based on clinical experience and narrative literature review rather than an exhaustive systematic literature review, and we cannot rule out the possibility that important risk factors were omitted. Moreover, this initial study population was restricted to individuals undergoing major, open, complex abdominal procedures within a single healthcare provider organization, factors that limit the generalizability of these findings to other types of procedures (e.g., orthopedic, neurosurgery) and populations from other geographic regions. With regard to model training and testing using a single 70%-30% split, there are always concerns for model overfitting. In future studies, other model validation approaches will include cross-validation or the creation of a second split to create a holdout dataset.

Despite being a well-documented risk factor for SSI, wound class (per the U.S. Centers for Disease Control and Prevention: I - clean; II - clean/contaminated; III - contaminated; and IV - dirty) [23] was not available in the EHR in this data sample and was excluded from the analysis [8,9]. Future efforts will explore the use of wound classification in SSI-PM.

## Conclusions

Two SSI predictive models - one that can be calculated manually and one that requires analytical resources - had high predictive accuracy. Use of either prediction model before the patient leaves the OR can enable proactive real-time intervention to mitigate risk and reduce SSIs. SSI risk prediction tools are an important component in a comprehensive approach to SSI reduction but will be limited in their effectiveness until they are seamlessly integrated into clinical workflow and enable real-time action at the point of care.
